# A New Player in Neuroblastoma: YAP and Its Role in the Neuroblastoma Microenvironment

**DOI:** 10.3390/cancers13184650

**Published:** 2021-09-16

**Authors:** Jenny Shim, Kelly C. Goldsmith

**Affiliations:** 1 Department of Pediatrics, Emory University School of Medicine, Atlanta, GA 30322, USA; jenny.shim@emory.edu; 2 Aflac Cancer and Blood Disorders Center, Children’s Healthcare of Atlanta, Atlanta, GA 30322, USA; 3 Winship Cancer Institute, Emory University, Atlanta, GA 30322, USA

**Keywords:** neuroblastoma, tumor microenvironment, yes-associated protein, hippo pathway, hypoxia, angiogenesis, extracellular matrix, metastasis, therapy resistance

## Abstract

**Simple Summary:**

Neuroblastoma is the most common extra-cranial solid tumor of childhood arising from the developing sympathetic neuroblast. Despite intense multimodal therapy, more than half of patients with high-risk neuroblastoma relapse with incurable disease. The Yes-Associated Protein (YAP) has been shown to play a critical role in many types of cancers, including neuroblastoma. YAP has also been recently highlighted as an important regulator of the tumor microenvironment (TME) that can affect cancer growth and response to therapies. Here, we focus on YAP and its role in neuroblastoma and the TME that underscores the therapeutic potential of inhibiting YAP in this highly aggressive pediatric solid tumor.

**Abstract:**

Neuroblastoma is the most common extra-cranial pediatric solid tumor that accounts for more than 15% of childhood cancer-related deaths. High risk neuroblastomas that recur during or after intense multimodal therapy have a <5% chance at a second sustained remission or cure. The solid tumor microenvironment (TME) has been increasingly recognized to play a critical role in cancer progression and resistance to therapy, including in neuroblastoma. The Yes-Associated Protein (YAP) in the Hippo pathway can regulate cancer proliferation, tumor initiation, and therapy response in many cancer types and as such, its role in the TME has gained interest. In this review, we focus on YAP and its role in neuroblastoma and further describe its demonstrated and potential effects on the neuroblastoma TME. We also discuss the therapeutic strategies for inhibiting YAP in neuroblastoma.

## 1. Introduction

Neuroblastoma is the most common extra-cranial solid tumor of childhood arising from the developing sympathetic nervous system [1], with approximately 800 cases per year identified in the United States [2]. Neuroblastoma is both a clinically and biologically heterogeneous cancer, with some children presenting with isolated or metastatic self-resolving disease, while other children present with widespread aggressive disease that is fraught with high morbidity and mortality [3]. Despite intensive therapy, including chemotherapy, surgery, myeloablative chemotherapy followed by autologous stem cell rescue, radiation, and immunotherapy, survival for patients with high-risk neuroblastoma remains poor [2], with approximately half of patients relapsing with aggressive chemotherapy-resistant disease [4]. Advances in understanding the genomic landscape and the tumor microenvironment (TME) in recurrent and newly diagnosed high-risk neuroblastomas has identified novel targets with therapeutic relevance in neuroblastoma patients [5,6]. For example, next-generation sequencing of paired diagnostic and recurrent high-risk neuroblastoma identified an increase in activating mutations in the RAS/RAF/MAPK pathway at relapse [7,8]. In addition to genomic alterations, gene set enrichment analysis of relapsed neuroblastoma tumors identified the downregulation of genes transcriptionally silenced by the Yes-Associated Protein (YAP), a core effector of the Hippo signaling pathway, compared to diagnostic tumors, suggesting increased YAP transcriptional activity at relapse [9]. In this particular study, increased YAP activation was noted to be the only event significantly associated with relapsed neuroblastoma [9]. The YAP/Hippo pathway has been well described to crosstalk with growth-promoting tumor-specific mutations, such as hyperactivating mutations in RAS/RAF/MAPK, which is further described in this review in the context of neuroblastoma. Therefore, the YAP/Hippo pathway may be a crucial target driving high risk neuroblastoma recurrence. Importantly, the complex neuroblastoma TME contributes to cancer progression [5,10,11,12,13,14,15,16,17] and YAP has been shown to regulate various aspects of the TME in many other cancer types [18,19,20,21,22,23,24,25,26]. Herein, we highlight YAP and its role in the neuroblastoma TME and how it may regulate therapy resistance as well as highlight opportunities for therapeutic YAP inhibition in this highly aggressive pediatric solid tumor.

## 2. YAP and Its Role in High-Risk Neuroblastoma

**Figure 1:**
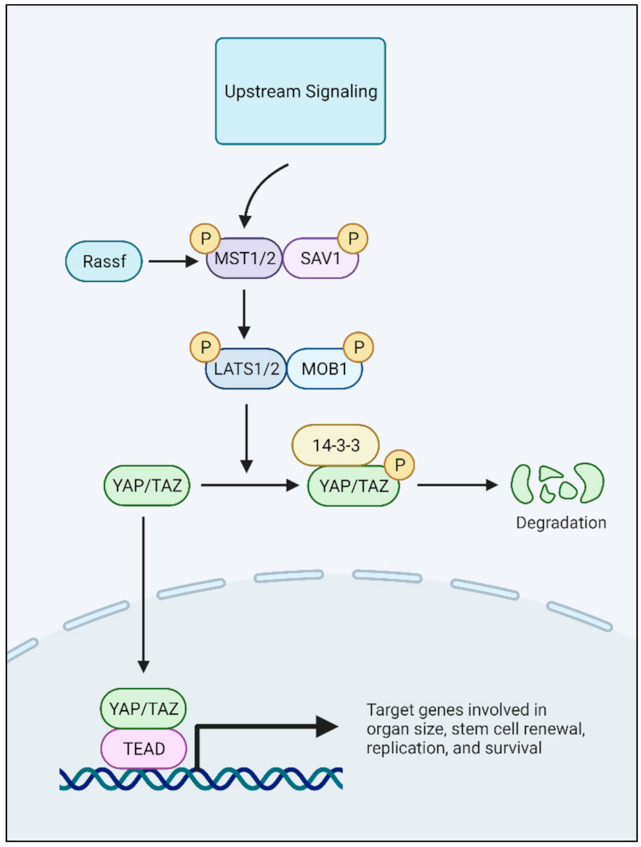
YAP/Hippo Signaling Pathway. Adapted from “Hippo Pathway in Mammals” by BioRender.com (2021). Retrieved from https://app.biorender.com/biorender-templates (accessed on 8 September 2021).

YAP is a transcriptional co-activator that binds with the TEA Domain (TEAD) family of transcription factors to initiate transcription of downstream target genes critical to organ growth and development [27,28,29,30,31,32]. The transcriptional co-activator with a PDZ-binding motif (TAZ) is a paralog of YAP. Both YAP and TAZ activity are regulated by the Hippo pathway kinases [32]. Hippo pathway proteins such as LATS1/2 and MST1/2 phosphorylate YAP and/or TAZ, leading to their cytoplasmic retention and ultimate degradation [29,32]. When the Hippo pathway is inactivated, YAP/TAZ can translocate into the nucleus and bind TEAD to regulate the transcription of effector genes (Figure 1). YAP has been reported to contribute to cell identity and tumor initiation, metastasis, angiogenesis, and resistance to chemotherapy in many solid tumors, such as head and neck, lung, colon, pancreas, and ovarian cancer [33,34,35,36,37,38,39,40]. YAP has also been reported to play a role in pediatric and young adult cancers, including rhabdomyosarcoma, osteosarcoma, Ewing sarcoma, and neuroblastoma [41,42,43,44,45,46]. 

In certain tumor types upon YAP inactivation, TAZ can independently bind to TEAD family members to compensate for YAP loss and regulate transcription [47,48]. Genetic inhibition of TAZ alone in neuroblastoma cells showed that TAZ may affect cell proliferation, self-renewal, and cell cycle progression independent of YAP expression in certain neuroblastoma models [49,50]. More recently, however, we and others have found no compensatory upregulation of TAZ expression or activity in response to YAP genetic inhibition [51,52]. Specifically, RNA sequencing of two neuroblastoma cell lines, SK-N-AS and NLF, both before and after YAP shRNA stable knockdown, showed that the expression of *WWTR1*, the gene encoding TAZ, decreases with *YAP* genetic inhibition. YAP/TAZ target genes follow a similar trend, confirming that TAZ is not being upregulated to balance the loss of YAP, supporting TAZ as an unlikely influence in neuroblastoma biology [51,52].

Research regarding YAP’s role in neuroblastoma supports that this Hippo pathway protein plays a role in almost every element of the “Hallmarks of Cancer”, many of which support a role for YAP in the TME (Table 1).

### 2.1. Tumorigenesis

YAP and TAZ in normal tissues transcriptionally control organ size and growth under normal physiologic conditions [60,61]. The Hippo proteins serve as an intrinsic regulator of organ growth such that when an organ reaches its optimal size, the Hippo cascade activates to sequester YAP/TAZ in the cytosol, leading to cessation of unfettered organ growth [30,60,61]. For example, in *Drosophila*, Yorkie (Yki; homolog of YAP in fruit flies) constitutive activation by inactivation of the Hippo cascade led to Yki nuclear accumulation and organ overgrowth, which was reversible when Yki expression was turned off [60,61]. Organ overgrowth occurred in murine models with similar unrestrained YAP activity that was reversed upon Hippo pathway activation [61]. Under malignant conditions, solid tumors capitalize on structural environmental cues normally upregulated during organogenesis or wound healing that downregulate Hippo proteins and upregulate YAP/TAZ; yet, concomitant oncogenic mutations in those tumors prevent the usual feedback inhibition of YAP, leading to uninhibited cell proliferation and tumor growth [62].

#### 2.1.1. Cell Proliferation

Multiple groups have investigated YAP and its effect on cell proliferation in neuroblastoma [51,53,54]. The results from these studies were mixed. Our group found no significant effect of YAP knockdown or YAP overexpression on neuroblastoma cell line proliferation in vitro [51]. Yang et al. and Shen et al. showed that siRNA and shRNA knockdown of YAP, respectively, decreased both cell viability and cell proliferation in neuroblastoma cells (SK-N-SH and SH-SY5Y) [53,54]. These conflicting results may be secondary to cell line characteristic/genetic differences and conditional properties such as metabolism and cell density that are known to influence YAP expression, especially in the setting of transient or incomplete YAP genetic inhibition [24,27,31,63]. For instance, the Hippo pathway downregulates YAP or inactivates YAP under high cell density conditions, leading to contact inhibition [31]. Moreover, glucose metabolism, which is very variable in high density culture conditions, has been shown to regulate YAP/TAZ interaction with TEAD, mediating YAP/TAZ/TEAD transcriptional activity [24,63]. Despite some inconsistency for its role in cell proliferation, a consensus finding is a decrease in colony formation in response to YAP knockdown, highlighting the clonogenic potential of cells with high YAP expression [51,53].

#### 2.1.2. Tumor Growth

YAP’s effect on neuroblastoma tumor growth in vivo seems to be significant and consistent, highlighting the collaborative role of YAP and the in situ tumor environment [51,55]. We have demonstrated that when SK-N-AS cells with stable YAP knockdown were injected subcutaneously into immunodeficient Nod *scid* gamma (*NSG*) mice, there was a significant delay in early tumor formation compared to control YAP-expressing SK-N-AS xenografts [51]. Seong et al. developed a metastatic neuroblastoma mouse model by injecting SK-N-AS cells into *NSG* mice via intracardiac injection and selected metastatic cells from the brain and bone marrow sites. Interestingly, the cells harvested from metastatic sites expressed higher YAP/TAZ protein compared to the parental cell line [55]. Importantly, shRNA knockdown of YAP/TAZ in the neuroblastoma cell lines derived from these metastatic subpopulations exhibited decreased tumor growth and prolonged disease-free survival in mice after intracardiac injection [55]. These results support that YAP may regulate unique cues in the TME that promote tumor survival as well as growth in neuroblastoma. 

### 2.2. Metastasis

Anoikis is a form of programmed cell death that occurs as cells remove themselves by apoptosis when they are not in the correct context of development within a tissue [64,65]. Cell detachment leads to Hippo pathway activation and YAP inactivation, eventually inducing anoikis [66]. Impaired anoikis with tumor cell survival can lead to enhanced tumor metastatic potential [64,65]. Anchorage-independent growth is a hallmark of cancer cells that have overcome anoikis [64,65]. YAP has been shown to contribute to anoikis resistance and metastasis in many cancers [36,40,67,68,69,70]. Indeed, in vitro studies showed impaired anchorage-independent growth and diminished invasion/migration abilities in neuroblastoma cells with YAP knockdown [56]. Moreover, YAP transcriptional activity and protein expression were increased in neuroblastoma patient-derived xenografts (PDXs) from patient tumors at relapse and from metastatic sites [51]. Furthermore, gene expression profiling from parental and metastatic subtypes of neuroblastoma cells developed in vivo from a metastatic mouse model revealed the Hippo signaling pathway to be enriched with YAP expression upregulated in metastatic populations [55]. YAP protein expression was elevated in metastatic cells compared to the parental cells and in PDX tumors derived from non-primary relapsed tumor compared to the diagnostic tumor [51,55]. Accordingly, YAP/TAZ knockdown suppressed the metastatic phenotype in vivo [55].

### 2.3. Therapy Resistance

The description of the detailed mechanisms underlying YAP-induced therapy resistance vary but they converge on one common theme: YAP/TAZ-mediated transcription is upregulated and responsible—either by the inactivation of upstream Hippo proteins or other negative YAP regulators or by direct YAP/TAZ activation [71,72,73,74]. While mutations in negative YAP regulators such as PTPN14 in relapsed neuroblastomas can lead to upregulated YAP transcriptional activity at relapse [9], not all YAP-expressing neuroblastomas harbor *PTPN14* mutations and the exact mechanism as to how YAP induces therapy resistance in neuroblastoma may be context-dependent based on the type of chemotherapy or targeted therapy used [71,72].

#### 2.3.1. Cytotoxic Therapy Resistance Imparted by YAP Differs In Vitro Versus In Vivo

We have shown that YAP expression and transcriptional activity immediately increase following one cycle of standard high risk neuroblastoma therapy, topotecan and cyclophosphamide, when given to mice harboring established neuroblastoma PDXs [51]. The tumors continued to grow through the treatment without significant regression. Given the brief exposure to therapy and sustained tumor growth, we posited that increased YAP expression post-therapy may represent a cell intrinsic response to the therapy itself rather than the selection of a YAP-expressing drug-resistant clone [51]. For instance, DNA-damaging agents, such as alkylating agents or topoisomerase inhibitors, have been reported to downregulate MST1 due to Hsp70-mediated degradation, or to downregulate micro RNAs that inhibit YAP translation [74,75,76,77]. By downregulating MST1 through Hsp-70-mediated degradation, LATS1/2 is inactivated, leading to YAP activation as YAP remains in its dephosphorylated state to enter the nucleus (Figure 1) [72]. In a hepatocellular carcinoma model, miR-590-50 was identified as a functional modulator of YAP, and YAP promoted chemotherapy resistance through the upregulation of genes involved in drug efflux pumps and stemness [76]. Thus, both Hippo-dependent and Hippo-independent mechanisms seem to affect therapy-induced expression of YAP in cancer.

In other cancer models, YAP has been shown to transcriptionally upregulate Bcl2 family pro-survival proteins, such as Bcl2 and Bcl-XL, to promote therapy resistance [78,79]. However, YAP knockdown does not affect Bcl2 family pro-survival protein expression in neuroblastoma [51]. Bim binding patterns to different pro-survival Bcl2 members determine Bcl2- or Mcl1-mediated apoptosis resistance in neuroblastoma [80]. Although we found that YAP knockdown upregulates the expression of the pro-apoptotic BH3 protein Bim, Bim remained sequestered to and inactivated by either Mcl1 or Bcl2, depending on the cell line tested—suggesting an alternative mechanism of cytotoxic therapy resistance by YAP occurs in neuroblastoma [51]. 

In a study exploring YAP and chemotherapy resistance in neuroblastoma, Yang et al. derived cisplatin-resistant SH-SY5Y cells and demonstrated that YAP siRNA inhibition led to reduced proliferation and colony formation in vitro while cells were still incubated in low dose cisplatin [53]. The cisplatin-resistant cells were likely re-sensitized to low dose cisplatin upon YAP knockdown, supporting YAP’s effect on cytotoxic therapy resistance. In contrast, we found that YAP knockdown or overexpression does not lead to significant sensitization or resistance, respectively, to cytotoxic agents such as melphalan in vitro [51]. Further, we noted no significant increase in apoptosis in response to etoposide in the setting of YAP knockdown in vitro. Contrastingly, SK-N-AS murine xenografts harboring shYAP tumors had significant tumor regression when treated with cyclophosphamide compared to YAP-expressing control tumors [51]. These findings support that YAP plays more of a critical role in therapy resistance within the TME verses 2D culture setting.

#### 2.3.2. Tyrosine Kinase Inhibitor Resistance Due to YAP

The dysregulation of Hippo signaling and subsequent activation of YAP/TAZ is a major resistance mechanism identified in response to multiple targeted therapies [71,72,73,74]. Two of the most studied targeted therapies in the context of YAP deregulation in cancers is EGFR and MAPK pathway inhibitors. Both EGFR and MAPK pathway signaling are critical for cell proliferation and survival; thus, oncogenic mutations in these pathways can lead to tumorigenesis [73]. YAP expression in lung cancer has been identified as the cause for drug resistance and tumor recurrence in response to EGFR tyrosine kinase inhibitors (TKI) [71,81]. Lee et al. noted upregulated YAP expression and activation in long-term TKI-induced resistant lung cancer cells and YAP inhibition of TKI-resistant cells restored sensitivity to TKI treatment [81]. The precise mechanism of how YAP drives TKI resistance in many cancers remains poorly understood, yet is thought to be secondary to YAP transcriptional target upregulation, such as *AXL*, *AREG*, *ERBB3*, and *CTGF* [82,83,84]. The RAS/MAPK pathway is frequently deregulated in many cancer types, including neuroblastoma, due to activating mutations in *ALK/RAS/RAF* or inhibitory *NF1* mutations or deletions [8,85,86,87]. Lin et al. reported that YAP promotes resistance to RAF- and MEK-inhibitors potentially through upregulation of the pro-survival gene *BCL-XL* and that combined YAP and RAF or MEK inhibition leads to synthetic lethality in *BRAF*- and *RAS*-mutant tumor types [88]. In rhabdomyosarcoma, YAP and oncogenic RAS cooperate in tumorigenesis, suggesting the importance of co-targeting these pathways [89]. Studies have shown that there may be cross-talk between YAP and the RAS/MAPK pathway: RAS pathway proteins stabilize YAP protein to prevent turnover and induction of the YAP downstream target AREG leads to EGFR/RAS pathway activation, forming a positive feedback loop [90,91]. 

Relapsed neuroblastoma tumors harbor increased RAS/MAPK pathway mutations compared to paired diagnostic tumors [8]. Neuroblastoma cells with RAS/MAPK pathway aberrations have shown sensitivity to MEK inhibitors trametinib and binimetinib in vitro [8,92]. However, in vivo treatment with single-agent binimetinib in various neuroblastoma cell line-derived xenograft models demonstrated inhibition of tumor growth and extended survival in *NRAS* or *NF1* mutated xenografts while *ALK* mutated tumors did not respond, likely due to persistent or alternative tyrosine kinase pathway activation [8,93]. Moreover, there may be a role for microRNAs such as the let-7 microRNA family for contributing to ALK inhibitor therapy resistance by regulating RAS expression [94,95]. Therefore, these findings suggest the need for combined targeting of pathways. Dual inhibition strategies, such as MEK inhibition in combination with BRAF, BET, and CDK4/6 inhibitors have been employed in neuroblastoma, but studies have reported limited anti-tumor activity or concerns of eventual escape and resistance [96,97,98,99].

Given the interaction between YAP and oncogenic RAS, Coggins et al. and our group investigated the combined effects of YAP genetic inhibition and MEK inhibition [51,52]. Coggins showed that the MEK inhibitor trametinib induces YAP nuclear translocation while reducing cytoplasmic YAP in *RAS-* or *NF1*-mutated neuroblastoma cell lines, suggesting resistance to MEK inhibitor therapy via YAP activation [52]. CRISPR-Cas9 knockout of YAP and constitutively active YAP expression promoted sensitization and resistance, respectively, to trametinib in neuroblastoma cell lines with *RAS* hyperactivation [52]. RNA sequencing of the *MYCN*-amplified NLF cells with and without YAP knockout treated with vehicle or trametinib showed that YAP mediates trametinib resistance through the transcriptional activation of *E2F* and *MYCN*, allowing the maintenance of the proliferative capacity of neuroblastoma cells [52]. Our studies expanded on this data, showing enhanced tumor regression in response to trametinib in an *MYCN* non-amplified *NRAS*-mutated SK-N-AS xenograft when YAP is genetically knocked down [51]. We further defined the mechanism for enhanced in vivo sensitivity to MEK inhibition, independent of *MYCN* amplification, which is further explained in Section 3.1.1 of this review.

### 2.4. Mesenchymal Properties

Cells with mesenchymal phenotypes exhibit extreme therapy resistance in cancer [100,101]. High YAP/TAZ activity has been observed in progenitor, or self-renewing, embryonic stem cells and is involved in the embryonic development of tissues and organs. YAP activation has also been shown to impair normal cell differentiation [102,103]. In the context of cancer, cancer stem cells (CSC) have been identified in neoplastic tissues and those that contain self-renewing and stem-like properties [103,104]. YAP/TAZ have been shown to participate in epithelial mesenchymal transition (EMT) and promote CSC self-renewal [37,103,105]. Moreover, the Hippo pathway is involved in developing neural tissue by preventing YAP/TAZ-driven neural progenitor growth and proliferation [106,107]. In fact, YAP promotes an early neural crest phenotype and displays a mesenchymal morphology [59]. Early neural crest cells are highly migratory with multipotential progenitor features, and therefore can give rise to sympatho-adrenal precursors as well as neuroblasts [108]. Thus, as a potential driver of the mesenchymal stem-like cell, YAP may induce therapy resistance.

#### 2.4.1. Neurosphere Formation

In pediatric and young adult cancer rhabdomyosarcoma, Slemmons et al. demonstrated that when rhabdomyosarcoma cells were cultured as “rhabdospheres” in neurobasal media, YAP and Notch expression were upregulated [44]. They further found that YAP suppression decreased expression of downstream stemness genes, *OCT4* and *SOX2*, in cells grown as rhabdospheres, suggesting the role of YAP in mesenchymal properties of embryonic tumors [44]. To investigate the mesenchymal properties of YAP in neuroblastoma, our group derived neurospheres from neuroblastoma cell lines in neurobasal media, mirroring the stem-like environment [51,109]. We noted increased YAP transcriptional activity along with increased *OCT4* and *SOX2* expression. YAP knockdown led to the suppression of mesenchymal gene expression despite neurobasal conditions, suggesting that YAP helps mediate the mesenchymal state [51]. We also noted fewer and an increased number of neurospheres in YAP knockdown and overexpressing neuroblastoma cells, respectively. To further support our findings, RNA sequencing of SK-N-AS cells with YAP shRNA knockdown verses control revealed the downregulation of genes involved in mesenchymal states in other tissue types, such as *JAK1*, *IL6ST*, and *TBX3* [110,111,112].

#### 2.4.2. Mesenchymal Phenotype

Neuroblastoma tumor cells demonstrate phenotypic heterogeneity. An increasing focus has been to fully characterize the mesenchymal and adrenergic lineages of neuroblastoma and to understand cell plasticity and the epigenetic regulation of these states [57,58]. Isogenic cell lines from the same patient distinguished solely by CD133+ (mesenchymal) and CD133- (adrenergic) phenotypes demonstrated extremely divergent mRNA profiles [58]. YAP and TAZ protein expression were consistently increased in the mesenchymal neuroblastoma cells and absent in the adrenergic populations [58]. In this study, the adrenergic and mesenchymal phenotypes were driven by distinct super-enhancer landscapes and super-enhancer-related transcription factors (TF), such as *PRRX1*, a TF that they mechanistically showed induces a mesenchymal state. These two states are able to interconvert, and YAP protein expression increased consistently following *PRRX1*-induced overexpression [57,58]. Importantly, the mesenchymal neuroblastoma cell types are more resistant to standard cytotoxic therapy compared to their adrenergic counterparts [58]. While recent studies have implicated that the *PRRX1* super-enhancer TF drives the mesenchymal state, in our hands, genetic knockdown of YAP alone leads to increased expression of adrenergic genes/proteins and decreased mesenchymal proteins in mesenchymal neuroblastoma cells. Reciprocally, YAP overexpression in an adrenergic neuroblastoma cell line leads to increase in SNAI2 and PRRX1 with a concomitant decrease in PHOX2B, GATA3, and DBH (data unpublished). Therefore, further exploration for whether YAP alone can drive the neuroblastoma mesenchymal phenotype is underway and may further support YAP as a critical therapeutic target in neuroblastoma. 

## 3. YAP and the Tumor Microenvironment in Neuroblastoma

### 3.1. Current Knowledge and Potential Contributions

**Figure 2:**
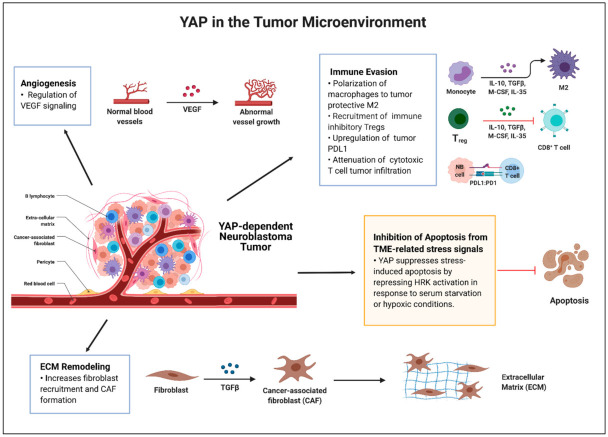
Potential roles for YAP in the neuroblastoma TME and areas for further investigation. YAP plays a role in every aspect of the TME from angiogenesis, ECM remodeling, immune evasion, and regulation of TME-related target genes, such as *HRK*. We have published on the novel relationship between YAP and HRK in neuroblastoma leading to regulation of stress-induced apoptosis in the tumor environment (yellow highlighted box). Areas of YAP TME regulation in other cancers that have also been described as TME factors in neuroblastoma support that further investigation should be pursued (blue boxes). Adapted from “The Tumor Microenvironment: Overview of Cancer-Associated Changes”, by BioRender.com (2021). Retrieved from https://app.biorender.com/biorender-templates (accessed on 8 September 2021).

Given the lack of recurrent driver mutations in diagnostic high-risk neuroblastoma, going beyond the genetic events and further understanding the neuroblastoma TME has been a crucial focus to identify novel therapies [16]. The TME in neuroblastoma has been extensively reviewed, pointing to roles for immune cells, non-immune cells, and *MYCN* amplification, influencing therapy response and survival [5,16,17]. Many studies highlight YAP as a regulator of key aspects of the TME that impact therapeutic response in cancers [23]. YAP tumor expression interacts with the TME to influence stress-induced apoptosis, tumor hypoxia, angiogenesis, extracellular matrix (ECM) remodeling, and the stromal and immune cell networks (Figure 2). These interactions ultimately impact tumorigenesis, metastasis, therapy resistance, and mesenchymal properties [23,113]. Therefore, further understanding the mechanisms underlying Hippo/YAP signaling in the TME in neuroblastoma may also provide therapeutic opportunities. 

The role of YAP in normal tissue wound healing and tissue regeneration is an important concept to understand how YAP may influence or be influenced by the neuroblastoma tumor environment [28,40,114]. We and others have shown that YAP protein expression and transcriptional activity increase as a response to chemotherapy in neuroblastoma tumors or in cells derived from metastatic sites [51,55]. Mutations in YAP/TAZ itself are rare and limited to certain cancer types; thus, recent investigations correlate YAP/TAZ oncogenic activation in solid tumors to “wounds that never heal”, as YAP is activated following extensive damage (due to radiation and/or chemotherapy), and in cooperation with oncogenic mutations driving proliferative pathways (such as RAS/MAPK, etc.), cooperate to drive a chronic regenerative response [40]. The lack of genomic YAP alterations (i.e., mutations, amplification) and our finding of increased YAP expression following a single cycle of cytotoxic therapy in *RAS*-mutated neuroblastoma in vivo, support that chemotherapy-induced damage may upregulate YAP in the tumor and may also explain the presence of increased YAP in post-chemotherapy relapsed primary neuroblastoma [51]. 

The chronic regenerative response includes YAP activation and pathways that suppress apoptosis, promote neo-angiogenesis, remodel the ECM, and recruit cancer-associated immune cells, forming a niche for cancer cell survival and proliferation. In the next section of the review, we will focus on the current literature highlighting YAP and its role in the TME and discuss potential contributing aspects of YAP in the neuroblastoma TME, especially in the context of stress-induced apoptosis, tumor hypoxia and vasculature, ECM remodeling, and the immune milieu.

#### 3.1.1. Tumor Environmental Stress-Induced Apoptosis

A tumor-promoting environment imparts signals that suppress stress responses to prevent cancer cell death in the face of nutrient deprivation or hypoxia. We found that YAP indeed can suppress stress-induced apoptosis in neuroblastoma [51]. Due to the significant impact of YAP inhibition in neuroblastoma xenografts that suppressed tumor growth and therapy responses in vivo, we investigated downstream targets regulated by YAP that might contribute to in situ tumor responses [51]. RNA sequencing data obtained from SK-N-AS cells with and without YAP knockdown showed that pathways involved in apoptosis, metabolism, and ECM remodeling, all processes important for the TME, were significantly affected. When evaluating the differential expression of genes in gene set enrichment analyses, we noted that *HRK*, a gene that encodes the protein Harakiri, was significantly upregulated in the cells with YAP knockdown [51]. 

Harakiri (HRK) is a BH3-only pro-death protein that activates the intrinsic or mitochondrial apoptosis pathway in the setting of cytokine deprivation and hypoxia, both properties in the solid tumor environment shown to promote therapy resistance [115,116]. We functionally validated HRK suppression by YAP in neuroblastoma cell lines, showed that HRK is suppressed when YAP is increased in relapsed tumors, and demonstrated that following chemotherapy treatment of PDXs in vivo, YAP expression increases while HRK expression decreases [51]. We were also able to restore cytotoxic therapy response in vitro in neuroblastoma cells by serum starvation, but apoptosis only occurred when YAP was genetically inhibited to allow for HRK expression and activation in response to serum deprivation and chemotherapy. Therefore, we identified HRK as a novel tumor suppressor in neuroblastoma that is negatively regulated by YAP to prevent therapy induced apoptosis in the in situ TME [51]. 

#### 3.1.2. Hypoxia and Angiogenesis

Hypoxia has been shown to induce YAP nuclear translocation and activation through inhibition of Hippo signaling [117,118]. In relation to the hypoxia-inducible factor 1 (HIF1) and its ability to drive glycolysis in the setting of hypoxia, YAP binds to HIF1 alpha, forming a complex that both sustains HIF1α stability and promotes glycolysis in hepatocellular carcinoma [26,117]. The hypoxic environment also leads to angiogenesis and YAP signaling is involved in tumor vasculature development [119]. Nuclear YAP/TAZ (active state) is expressed in developing endothelial cells (ECs) and the remodeling vasculature [120,121]. Moreover, recent studies demonstrated that YAP activation is crucial for angiogenesis regulated by VEGF signaling and cytoskeletal remodeling [122]. Active EC YAP induces a downstream transcriptional program which regulates further ECM remodeling for a tumor niche [20]. Thus, therapeutic targeting of YAP may be important for vascular normalization to improve cancer-directed therapies.

In neuroblastoma, hypoxic conditions can shift cells into a de-differentiated or stem-like phenotype [123]. Jogi et al. showed that neuroblastoma cells in hypoxic conditions were shifted towards an immature, neural crest-like phenotype with an increase in gene markers of neural crest sympathetic progenitors [124]. Patients with hypoxic tumors were predicted to have an unfavorable prognosis with tumors associated with telomerase activation and a more immunosuppressive, poorly differentiated, and apoptosis-resistant TME [125]. Angiogenesis occurs through angiogenic factors such as vascular endothelial cell growth factor (VEGF), platelet-derived growth factor (PDGF), and fibroblast growth factor (FGF) and are influenced by hypoxia and inflammation [5,16]. HIF1 transcription factors and *MYCN* have been shown to be involved in both processes [16,126]. In addition to hypoxia, increased VEGF expression and tumor angiogenesis correlates with more aggressive disease and poor outcomes in neuroblastoma [127]. Therefore, the role for YAP as well as HRK in hypoxia and angiogenesis in neuroblastoma deserve further exploration.

#### 3.1.3. Extracellular Matrix Remodeling

The ECM affects and is affected by tumor cells bidirectionally, acting as the signaling hub and organizer for tissue homeostasis [113,128]. The ECM is comprised of a complex nest of structural proteins, such as collagen, hyaluronic acid, elastin, matricellular proteins, glycoproteins, proteoglycans, and polysaccharides. These molecules form a dynamic and versatile network of a cell-matrix environment that form the structural foundation for tissue function and mechanical sustainability [128,129]. Abnormal ECM dynamics can contribute to cancer development and progression by promoting a niche for cancer cells to metastasize and invade surrounding tissues [129,130,131]. In cancers, hypoxia and inflammation in the TME can promote ECM stiffness due to an increase in collagen deposition, leading to upregulation of integrin signaling and various pathways including PI3K/AKT to promote cell proliferation and anti-apoptotic effects [131]. YAP/TAZ and their role in mechanotransduction is well described [20,132]. YAP and TAZ act as mechanotransducers and sensors of mechanical cues from the ECM and receive and communicate those signals in a bidirectional manner. YAP and TAZ can be induced by increased ECM rigidity or stiffness in the setting of inflammation and tissue damage [20,23]. Cells spreading over a surface can activate YAP/TAZ as well. Furthermore, YAP/TAZ activity requires the GTPase Rho, which regulates the actin cytoskeleton and cytoskeletal tension induced by the pulling forces of the ECM [20,132]. Most importantly, YAP and TAZ are required mediators of ECM elasticity and cell geometry, as alterations of YAP/TAZ levels can overrule cell mechanophysical behavior [20]. YAP/TAZ activation can remodel the ECM itself through complex pathways to promote cancer aggressiveness, metastasis, and therapy response [23]. A recent study by Jang et al. demonstrated that matrix stiffness epigenetically regulates YAP activation in gastric cancer through DNA methylation modifiers leading to YAP promoter hypomethylation, proposing that epigenetic reprogramming of the ECM properties in solid tumors may be a potential therapeutic strategy [133]. 

In neuroblastoma, Lam et al. artificially increased the rigidity of polyacrylamide hydrogels on which neuroblastoma cells were seeded to mimic increased stiffness of the ECM. They showed that increased ECM rigidity enhanced neuritogenesis (measurement of differentiation), decreased proliferation, and reduced expression of *MYCN* [134]. Additionally, retinoic acid, which is a differentiating agent currently used in the clinical setting of high risk neuroblastoma therapy, potentiated neuroblastoma differentiation with increasing ECM stiffness [134]. More studies are needed to validate these findings in the context of YAP. If changes in ECM stiffness influences differentiation, *MYCN* expression, and YAP expression, then the use of therapies focused on remodeling the ECM may have therapeutic gain in neuroblastoma. Indeed, therapies targeting ECM components are heavily under investigation as a novel anticancer approach [128,135]. Unpublished data from our laboratory demonstrate that neuroblastoma cells cultured in low density express increased YAP compared to high density states, suggesting YAP is influenced by contact inhibition in addition to ECM rigidity states. Therefore, YAP and its role in mechano-sensing is complex and ECM remodeling strategies to target structural components or signaling molecules and remodeling enzymes deserve further investigation in the context of YAP in neuroblastoma [130,135].

#### 3.1.4. Immune Milieu

In the era of immunotherapy, understanding the immune milieu in the TME has been important to strategize ways to improve immunotherapy response. As described in depth by S. Joshi and Blavier et al., stromal cells (cancer-associated fibroblasts (CAFs) and mesenchymal stromal cells (MSCs)) and immune cells (tumor-associated macrophages (TAMs), myeloid-derived suppressor cells (MDSCs), regulatory T cells (Tregs), and tumor infiltrating lymphocytes (TILs)) are all key players of the neuroblastoma TME [5,17]. YAP’s role in the immune environment in other cancers has been described. YAP can activate CAFs, establishing a feed-forward loop, leading to a cancer-friendly niche with more ECM rigidity [23,136]. Through the transcriptional regulation of cytokines and chemokines, YAP has been shown to influence the phenotype of tumor-resident immune cells in favor of an inhibitory environment [23,137]. For example, YAP activation is associated with TAM recruitment and M2 phenotype polarization, leading to a pro-tumorigenic or immune suppressive environment, potentially via C-C motif chemokine ligand 2 (CCL2 or MCP1) activation, as shown in hepatocellular carcinoma [138,139,140]. YAP also contributes to the immune-suppressive environment further by recruiting MDSCs to suppress cytotoxic T cells through the production of cytokines such as IL-6, macrophage colony-stimulating factor (CSF1), and granulocyte–macrophage colony-stimulating factor (GM–CSF) in pancreatic and prostate cancers [141,142]. Additionally, Stampouloglou et al. and Lebid et al. evaluated YAP expression in activated CD4+ and CD8+ T cells and found that the loss of YAP in T cells resulted in enhanced T cell activation, differentiation, and function, leading to improved T cell infiltration in tumors, signifying that YAP therapeutic inhibition in immune cells themselves may contribute to improved immunotherapy responses [143,144]. Tregs are important for immune homeostasis, and YAP expression in these cells has shown to be essential to suppress anti-tumor immunity [21]. Recent studies have also reported that YAP affects immune check points, by upregulating programmed cell death ligand 1 (PDL1) expression on tumor cells to turn off tumor-specific effector T cells and escape antitumor immunity [145,146]. Overall, YAP’s role specifically in the pediatric cancer immune environment remains to be explored.

### 3.2. Future Investigations

YAP has been shown to promote a tumor permissive environment through inhibiting TME stress-induced apoptosis, remodeling the ECM and vasculature, and suppressing the immune response in other cancers. Although we have identified a novel interaction in neuroblastoma—YAP-mediated repression of the tumor suppressor HRK—that could explain one of the roles for YAP in the context of tumor environmental stress-induced apoptosis, further studies are needed to fully understand the breadth of YAP’s regulation in the neuroblastoma TME. Figure 2 summarizes the ways in which YAP contributes to the general TME (highlighted in the blue box) and in neuroblastoma specifically (highlighted in the yellow box) and areas in which further investigations are warranted based on the role of YAP in other cancers. We understand that all tumors and their genetic/epigenetic environment and the interplay amongst signaling pathways are complex and different, supporting these interactions cannot be inferred but must be explored in neuroblastoma specifically. 

Importantly, the neuroblastoma TME is dynamic and complex [5,17]. As shown in Figure 2, YAP is involved in almost every aspect of the solid tumor environment that composes the neuroblastoma TME. We also note that the interaction between the TME and YAP is bidirectional, suggesting the oncogenic role of YAP in promoting a pro-tumorigenic environment can further potentiate YAP activation or related pathways. For instance, YAP is activated by the stiff and rigid ECM formed by CAFs, which in turn leads to downstream signaling of further ECM remodeling, creating a cancer niche suitable for metastasis and tumor growth [23,132]. 

While our studies have identified YAP’s role in suppressing HRK to promote neuroblastoma tumor growth and resistance to chemotherapy and targeted therapy in the in situ tumor environment [51], further investigations in our laboratory are underway to understand the actual mechanism for how YAP regulates HRK and other tumor suppressor genes to inhibit their expression and activity. Whether this mechanism or others leads to YAP influences on immunotherapy responses in neuroblastoma is also a topic of heavy exploration in our laboratory.

YAP has been in the spotlight over the past few years for its role in the ECM organization and mechanotransduction, especially in the context of cancer [20,132]. Therefore, further studies using models that recapitulate the neuroblastoma ECM in the context of YAP may help delineate other pathways and signaling events that promote the tumor niche for growth and metastasis. In addition to its direct role in ECM organization and mechano-sensing, YAP can promote CAFs to induce a stiffer ECM for tumor growth. CAFs have been identified in neuroblastoma tumors as well and are associated with a poor outcome due to a more therapy-resistant phenotype [17,147], supporting the need to explore YAP’s contributions to neuroblastoma CAF formation.

YAP and its role in the cancer immune milieu has been surfacing with advances in immunotherapy. The emerging role of YAP in the cancer immune environment is an ongoing area of research to improve immunotherapy approaches. Thus, further understanding YAP’s role in the neuroblastoma immune environment may provide the opportunity to improve targeted immunotherapy responses. Many groups have begun to characterize the neuroblastoma immune landscape and ongoing studies continue to demonstrate low immunogenicity, low T and NK cell tumor infiltration, and immune evasion strategies [148,149]. Further investigations in our laboratory are being pursued to understand YAP’s impact on the neuroblastoma immune environment and response to immunotherapies. Collectively, studies exploring the mechanisms behind YAP and neuroblastoma TME regulation will be essential to identifying therapeutic targets and pathways that either cooperate with or are able to antagonize YAP’s oncogenic effects in neuroblastoma. 

Finally, we would like to emphasize and highlight the importance of using models that closely recapitulate the neuroblastoma TME, given the strong influence each aspect of the TME (ECM stiffness, cellular contact, EC interactions) has on YAP expression. A variety of pre-clinical models for studying neuroblastoma exist, ranging from in vitro 2D culture to 3D bioprinted models that strive to recapitulate the in vivo setting. Multiple recent publications and reviews have illustrated the emerging development and use of 3D in vitro models for pre-clinical studies in neuroblastoma [150,151,152,153,154]. In fact, 3D bioprinted models have the advantage of high tunability with the addition or removal of TME components such as immune or stromal cells and manipulation of vasculature or ECM properties. However, studies have shown that in vivo models, especially PDX models and immunocompetent transgenic or humanized mouse models, most closely resemble the neuroblastoma TME, and as such serve as an important tool for validating in vitro findings [150,155]. For example, future studies exploring YAP genetic knockout in the *TH-MYCN* transgenic mouse or *MYCN* amplified zebrafish models of neuroblastoma may contribute to our understanding of YAP and *MYCN* and their TME effects [156,157]. 

## 4. Therapeutic Targeting of YAP in Neuroblastoma

There are various published studies and reviews regarding the Hippo pathway and YAP/TAZ targeting [114,158]. Many direct and indirect inhibitors targeting YAP and related pathways have been described. We have summarized potential therapies to target YAP and related pathways in Table 2. Verteporfin, a photodynamic therapy that is FDA-approved for macular degeneration, has been shown to disrupt the YAP-TEAD complex by directly inhibiting YAP and has been the most widely used “YAP inhibitor” in pre-clinical studies of YAP driven cancers [159,160]. Unfortunately, we and other groups have found off-target and non-specific cytotoxicity induced by verteporfin specifically in YAP null neuroblastoma cells, likely through the activation of reactive oxygen species [161]. Other groups have looked at cyclic peptides that disrupt the YAP-TEAD binding pocket, yet no significant pre-clinical evidence has validated their clinical use [162,163]. Additionally, we have found that YAP mimetic peptides are ineffective due to poor cell membrane penetrability and high potential for being protein-bound (data unpublished). Small molecules that inhibit TEAD auto-palmitoylation, a post-translational modification essential for TEAD activation and binding to YAP, pre-clinically show efficacy in *NF2*-mutated malignant mesothelioma and meningiomas or schwannomas, leading to a first in human phase 1 clinical trial in those tumor types, supporting the need to assess their efficacy in YAP-driven neuroblastoma [164,165,166]. 

In addition to YAP-TEAD directed therapy, combination therapies targeting YAP downstream effectors and its cooperative pathways are critical avenues to explore. Currently, how YAP regulates HRK expression in neuroblastoma is unknown. HRK is inactivated in other cancers via epigenetic silencing through DNA promoter hypermethylation or histone modifications [167,168,169,170]. YAP-TEAD has also been shown to impart chromatin alterations on target gene loci to induce or repress target gene expression in other cancers [171,172,173,174]. Therefore, epigenetic modifying agents may have therapeutic potential in the context of HRK upregulation to restore stress-induced apoptosis in neuroblastoma. Specifically, the histone deacetylase (HDAC) inhibitor vorinostat has already shown pre-clinical efficacy in potentiating anti-tumor therapy effects in early-phase clinical trials in combination with isotretinoin or I-131 MIBG therapy in neuroblastoma [175,176]. In *RAS*-mutated melanoma and non-small cell lung cancer, where YAP also plays a significant role, the combination of HDAC inhibitors and MEK inhibitors has shown in vivo antitumor effects [177,178]. Thus, considering combination therapies with epigenetic modifying agents (DNA demethylating agents or HDAC inhibitors) and those that target YAP-cooperating pathways in the TME (MEK inhibitors or BET inhibitors) could be considered. Investigations are underway in our laboratory to define YAP’s method for HRK and other gene suppression and preclinically target those pathways with similar novel combinations. Until a sensitive and specific YAP inhibitor is developed and identified for its therapeutic potential in neuroblastoma, alternative methods to exploit targets and signaling pathways downstream of YAP should continue to be explored. 

## 5. Conclusions and Future Directions

In this review, we have provided a comprehensive summary of the oncogenic role for YAP in neuroblastoma. Further, we outlined YAP and its influence on surrounding cells and stroma that sculpt the complex TME, and the potential effects that YAP may impart on the high risk neuroblastoma TME, specifically in the setting of stress-induced apoptosis, neo-angiogenesis, ECM remodeling, and the immune milieu. Most importantly, we propose ways to further investigate YAP in the neuroblastoma TME for the discovery of novel therapeutic opportunities. Overall, the presented data and literature support YAP as a logical therapeutic target in high risk neuroblastoma. While the implementation of the first phase 1 trial of a high affinity TEAD inhibitor for adult cancers gives promise that targeting YAP/TEAD therapeutically now has higher potential, results from this review support that a combinatorial approach will be most optimal for YAP-driven heterogeneous tumors, such as neuroblastoma, that carry cooperating alterations that may attenuate single-agent TEAD inhibitor potency. Therefore, future investigations are critical to understanding the mechanisms underlying the role of YAP in the neuroblastoma TME and to identify optimal therapeutic strategies to target YAP directly or indirectly in novel combinations to improve outcomes for patients with high risk and relapsed neuroblastoma. 

## Figures and Tables

**Table 1:** YAP in neuroblastoma: summary of the literature.

Focus	Comments	References
Tumorigenesis	YAP knockdown decreases cell proliferation and colony formation in neuroblastoma cells YAP genetic knockdown demonstrates significant delay in neuroblastoma tumor growth in vivo	[51,53,54,55]
Metastasis	YAP/Hippo pathway is identified in metastatic subtypes of a metastatic neuroblastoma mouse model YAP expression is higher in neuroblastoma tumors derived from metastasis compared to primary tumor Inhibition of YAP/TAZ decreases the metastatic potential of cells derived from metastatic sites Invasion and migration abilities are impaired in neuroblastoma cells with YAP knockdown	[51,55,56]
Therapy Resistance	YAP knockout sensitizes neuroblastoma cells to MEK inhibition and YAP overexpression imparts MEK inhibitor resistance YAP knockdown sensitizes neuroblastoma xenografts to cytotoxic or MEK inhibitor therapy in vivo YAP maintains cisplatin resistance in neuroblastoma cells	[51,52,53]
Mesenchymal Properties	YAP is identified as a mesenchymal marker in CD133+ mesenchymal-type neuroblastoma cells YAP promotes neuroblastoma neurosphere formation and regulates stemness genes YAP promotes an early neural crest phenotype and migration in neuroblastoma cells	[51,57,58,59]

The table summarizes a review of the recent literature describing the function of YAP in neuroblastoma.

**Table 2:** Potential therapies to target YAP and related pathways.

Therapy	Mechanism and Effects	References
Verteporfin	Disrupt YAP-TEAD interaction Non-specific cytotoxic effects in neuroblastoma	[159,160,161]
Cyclic Peptides (i.e., Peptide 17)	Disrupt YAP-TEAD binding pocket No significant pre-clinical evidence in neuroblastoma	[162,163]
TEAD auto-palmitoylation inhibitors	Inhibit TEAD auto-palmitoylation and prevent YAP-TEAD binding Effect seen in *NF2*-mutated malignant mesothelioma cells and now in phase 1 clinical trials in adults	[166,179]
Combination therapies	HDAC inhibitors, such as vorinostat, with isotretinoin or I-131 MIBG in neuroblastoma MEK inhibitors in combination with HDAC inhibitors in *RAS*-mutated melanoma and non-small cell lung cancer HDAC inhibitors with anti-angiogenic agents or ECM-remodeling agents in solid tumors	[113,135,175,176,177,178]

The table summarizes potential YAP/TEAD inhibitors or related YAP pathway targeted therapy approaches.
